# Supplementary material for: Causal Relationship between Aging and Anorexia Nervosa: A White-Matter-Microstructure-Mediated Mendelian Randomization Analysis
Source: Biomedicines. 2024 Aug 16;12(8):1874. doi: 10.3390/biomedicines12081874 (PMC11351342; doi:10.3390/biomedicines12081874)
Supplement: Supplementary file 1 [file biomedicines-12-01874-s001.zip › Supplementary Figure/Figure S8/Leaveoneout plot of MR analysis for TL to ubm-b-2074.pdf]

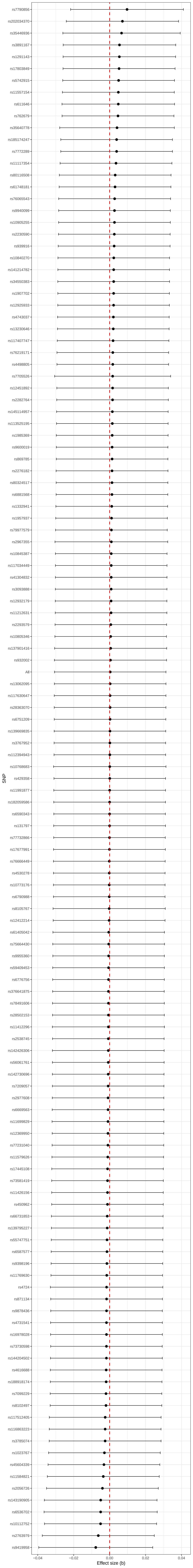

rs7790856
rs202034370
rs35446936
rs3891167
rs1291143
rs17803849
rs5742915
rs11557154
rs611646
rs762679
rs35640778
rs185174247
rs7772289
rs11117354
rs80116508
rs61748181
rs76065543
rs9940099
rs10905255
rs2230590
rs939916
rs10840270
rs141214782
rs34550383
rs1907702
rs12925933
rs4743037
rs13230646
rs117407747
rs76219171
rs4498805
rs7705526
rs12451892
rs2282764
rs145114957
rs113525195
rs1985369
rs9600019
rs869785
rs2276182
rs80324517
rs6881568
rs1332941
rs1957937
rs79977579
rs2967355
rs10845387
rs117034449
rs41304832
rs3093888
rs12932179
rs11212631
rs2293579
rs10805346
rs137901416
rs932002
A8
rs13062095
rs117630647
rs28363070
rs6751209
rs139669835
rs3767952
rs112394943
rs10768683
rs429358
rs11991877
rs182059586
rs6590343
rs131797
rs77732866
rs17677991
rs76666449
rs4530278
rs10773176
rs6790988
rs8105767
rs12412214
rs61405042
rs75664430
rs9955360
rs59409453
rs6776756
rs376641875
rs78491606
rs28502153
rs11412296
rs2538745
rs142426306
rs56061761
rs142730696
rs7209057
rs2977608
rs6669563
rs11699829
rs12369950
rs77231040
rs11579626
rs17445108
rs73581419
rs11426156
rs450962
rs66731853
rs139795227
rs55747751
rs6587577
rs9398196
rs11769630
rs4724
rs871134
rs9878436
rs4731541
rs16978028
rs73730598
rs144204502
rs4616688
rs188918174
rs7099229
rs8102497
rs117512405
rs116863223
rs3785074
rs1023767
rs45604339
rs11584821
rs2056726
rs143190905
rs6536702
rs10112752
rs2763979
rs9419958
SNP
-0.04
-0.02
0.00
0.02
0.04
Effect size (b)
